# In Silico Drug Design and Discovery: Big Data for Small Molecule Design

**DOI:** 10.3390/biom13010044

**Published:** 2022-12-26

**Authors:** Carmen Cerchia, Antonio Lavecchia

**Affiliations:** “Drug Discovery” Laboratory, Department of Pharmacy, University of Naples Federico II, 80131 Naples, Italy

Across life sciences, the steadily and rapidly increasing amount of data provide new opportunities for advancing knowledge and represent a key driver of emerging technological advancements. These data, some of which are available in the public domain, can be generated in different settings, for instance by experimental work or by computations. Therefore, it is crucial to make the best possible use of this plethora of information to improve decision-making. In light of this, there is increasing interest in computational methods to take advantage of this volume of data, including data mining and visualization methods, artificial intelligence and machine learning algorithms [1,2,3]. The aim of this Special Issue is to showcase recent applications of in silico approaches, making use of data from different domains, to support different aspects of drug design and discovery. This Issue includes five research articles and a review. 

The article by Chávez-Hernández et al. reported an innovative chemoinformatic protocol for de novo design of a virtual compound library of putative HIV-1 protease inhibitors [4]. The library was enumerated by using natural product fragments extracted from the COCONUT database [5], currently the largest collection of natural products. The obtained compounds showed reasonable synthetic feasibility and ADME-Tox properties, similar to some FDA-approved HIV-1 protease inhibitors. The authors envisaged the chemical synthesis and experimental screening of selected compounds as a future research direction. Interestingly, the chemoinformatic platform can be generalized and can also be adapted to different therapeutic targets.

Nasser et al. proposed a novel strategy for the identification of relevant patterns in molecules by using an autoencoder (AE) [6]. An AE is a deep learning (DL) architecture based on three main components: an input layer to be fed with input data, hidden layer(s) and an output (decoding) layer. The main advantage of AEs is the ability to handle low-dimensional feature representation from the inputs while preserving significant underlying features, in this case for molecular dimensionality reduction. The method essentially aimed to enhance the similarity search by eliminating unnecessary and redundant molecular features. The overall performances were judged using different metrics, including the Tanimoto similarity, adapted similarity measure of text processing and quantum-based similarity method.

Zajec et al. applied a structure-based virtual screening to discover inhibitors able to target an allosteric binding site on the C-terminal domain (CTD) of Hsp90 [7]. This is a relevant target for the development of anticancer agents, since it is overexpressed in many cancers, thereby promoting carcinogenesis by correctly folding oncogenic proteins such as c-Raf, Her2, Akt, HIF1 and CDK. One of the selected hit compounds, TVS-23, showed antiproliferative activity with an IC_50_ value of 26.4 ± 1.1 µM in the MCF-7 breast cancer cell line. This compound was optimized through the design and synthesis of structural analogues. Among them, 7l turned out to be the most potent, with IC_50_ = 1.4 ± 0.4 µM in MCF-7 and IC_50_ = 2.8 ± 0.4 µM in SK-N-MC Ewing sarcoma cell lines. The structures of the Hsp90-TVS23 and Hsp90-7l complexes underwent extensive molecular dynamics (MD) simulations of 1000 ns and the resulting trajectories were used for pharmacophore feature analysis, yielding a total of 5000 structure-based pharmacophore models. This analysis allowed the rationalization of the structural basis of the enhanced potency of 7l with respect to TVS-23.

Spinozzi et al. developed a freely available web service, SiCoDEA (Single and Combined Drug Effect Analysis), for the estimation of the potential synergistic, antagonistic or additive effects of drug combinations [8]. The platform is based on the software R, with the underlying models providing a combination index (CI) indicating synergy (CI < 1), antagonism (CI > 1) and additivity (CI = 1). The authors reported an exemplary application for the analysis of acute myeloid leukemia (AML) cells harboring the nucleophosmin (NPM1) mutation, which is the most frequent form of AML in adult patients. In particular, they evaluated the anti-proliferative effect of Homoharringtonine (HHT, also named Omacetaxine mepesuccinate) and ABT-199 (Venetoclax) on AML cell lines, indicating that such a combination has a synergistic effect in the mutated AML cell line.

The work by Guzelj et al. described the first modulators of nucleotide-binding oligomerization domain-containing protein 2 (NOD2) identified by virtual screening [9]. NOD2 plays an important role in the development of innate and adaptive immunity because it recognizes bacterial peptidoglycan fragments. Of note, the immune response mediated by NOD2 has also been associated with atherosclerosis. The structure-based design of NOD2 modulators has been limited by the absence of a structure of NOD2 in complex with a ligand. Therefore, the authors first obtained a homology model of human NOD2 using the NOD2 structure from rabbits as a template. Then, a hybrid docking–pharmacophore modelling strategy was implemented, in which the docked poses of previously reported NOD2 ligands were used to generate pharmacophoric hypotheses. This procedure allowed the identification of two compounds, 1 and 3, displaying inhibitory effects on NOD2-activation signaling triggered by MDP and SG8, two potent NOD2 agonists belonging to the muropeptide and desmuramylpeptide structural classes. However, additional assays would be required to confirm that the observed effects are due to a direct interaction of these compounds with NOD2 or with downstream signaling proteins.

The review by Pereira and Vale discussed potential repurposing opportunities for the HIV protease inhibitor saquinavir [10]. First, they extensively described saquinavir’s mechanism of action, pharmacokinetic properties and metabolism. Then, the authors discussed some reports of saquinavir’s inhibitory activity on SARS-CoV-2 proteins, such as the main protease (3CLpro) or RNA-dependent RNA polymerase (RdRp), but most of the results were obtained in silico. Finally, they discussed several studies in which saquinavir was reported to inhibit cell invasion, to enhance radiosensitivity and also to induce cytotoxicity and apoptosis in different types of cancer, both in vitro and in vivo. However, saquinavir never advanced to clinical trials for cancer treatments.

In conclusion, the contributions collected within this Special Issue highlight the central role of data, which represent the foundation of every model. Data quality, availability and management are, in fact, essential for accurate and reliable predictions. Therefore, we can foresee that the implementation of best practices for data handling will play an increasingly important role in maximizing the value of data and expediting innovation in the future.
